# The Value of CEUS in Distinguishing Cancerous Lymph Nodes From the Primary Lymphoma of the Head and Neck

**DOI:** 10.3389/fonc.2020.00473

**Published:** 2020-04-21

**Authors:** Ji Nie, Wenwu Ling, Qianru Yang, Hongyu Jin, Xuejin Ou, Xuelei Ma

**Affiliations:** ^1^Department of Biotherapy, State Key Laboratory of Biotherapy and Cancer Center, West China Hospital, Sichuan University and Collaborative Innovation Center of Biotherapy, Chengdu, China; ^2^West China School of Medicine, Sichuan University, Chengdu, China; ^3^Department of Ultrasound, West China Hospital, Sichuan University, Chengdu, China; ^4^Department of Liver Surgery, West China Hospital, Sichuan University, Chengdu, China; ^5^Department of Oncology, West China Hospital, Sichuan University, Chengdu, China

**Keywords:** contrast-enhanced ultrasonography, CEUS, lymphoma, cancerous lymph nodes, cervical lymph nodes

## Abstract

**Aim:** The purpose of this study was to assess the ability of contrast-enhanced ultrasonography (CEUS) in the differential diagnosis of cancerous lymph nodes.

**Methods:** Contrast-enhanced ultrasonography was performed in the cervical nodules of included patients, and the diagnoses were confirmed by pathological examination. Contrast-enhanced ultrasonography images and parameters of head and neck lymphomas were compared with those of cancerous lymph nodes. Besides, receiver operating characteristic curve was operated to access the diagnostic value of CEUS.

**Results:** Finally, a total of 63 head and neck lymphomas and 80 cervical cancerous lymph nodes were enrolled in this study. Results showed that the CEUS images of lymphoma were mainly characterized by homogeneous enhancement (71.43%), and approximately half of them were centripetal perfusion (58.73%), whereas most CEUS images of cancerous lymph nodes were inhomogeneous enhancement (82.50%) and centripetal perfusion (92.50%). Quantitative analysis of CEUS parameters indicated that PI (derived peak intensity) and AUC (area under the curve) of lymphomas were both lower than those of cancerous lymph nodes (PI: 8.78 vs. 10.51, AUC: 652.62 vs. 784.09, respectively) (*P* < 0.05). Receiver operating characteristic analysis showed that the sensitivity of CEUS parameters in the differential diagnosis was significant (80.00%), although the specificity was not high (47.62%). When parameters were combined with the image features, the accuracy of diagnosis was greatly improved (from 0.655 to 0.899).

**Conclusion:** Contrast-enhanced ultrasonography could be a promising tool for the differential diagnosis of head and neck lymphomas and cancerous lymph nodes.

## Introduction

Lymphomas, malignant diseases that originate from lymph nodes or lymphoid tissues, are normally divided into Hodgkin and non-Hodgkin lymphoma (1). The prognosis of lymphoma is poor, which is related to the fact that most patients are already in advanced stage when diagnosed. Most lymphoma patients display no obvious clinical symptoms at early stage, and sometimes cervical lymphadenopathy is the only initial complaint (2). Therefore, it is very important to determine the nature of cervical lymphadenopathy for the diagnosis and prognosis of head and neck lymphoma. Theoretically, there are various diseases that may contribute to lymph node enlargement in the neck, including head and neck cancer, infection, tuberculosis, and so on (3). Thus, early diagnostic methods with high sensitivity to cervical lymphadenopathy are extremely important.

The diagnosis of lymphadenopathy depends mainly on histological biopsy, which is considered as the gold standard (4). However, very little tissue is obtained and examined at biopsy, which sometimes makes it difficult to confirm the diagnosis (5, 6). Besides, staging is critical for determining treatment strategy and accessing disease prognosis, and information afforded by biopsy is not enough for accurate stage (7). Therefore, imaging examination plays a pivotal role in the diagnosis of malignant lymphoma.

There are many imaging methods for the diagnosis of cervical lymphadenopathy, including ultrasound, enhanced computed tomography (CT), enhanced magnetic resonance imaging, and positron emission tomography/CT (7–10). Among them, conventional ultrasound is the most preferred imaging modality because it provides many vital parameters of lymph nodules, including size, morphology, and internal structure (11). However, ultrasonographic morphology of lymphoma often overlaps with that of cancerous lymph node, which makes it hard to distinguish them by conventional ultrasound (12).

Contrast-enhanced ultrasonography (CEUS), combined with the use of ultrasound contrast agent, is capable of providing a real-time visualization of microvascular condition and blood supply for lymph nodes. In addition, with the help of time–intensity curve (TIC), quantitative analysis of blood perfusion in the lesions provides more diagnostic information for lymph nodes (13). Therefore, CEUS may play a role in distinguishing between head and neck lymphoma and malignant lymph nodes. So far, there have been numerous studies that investigate the efficiency of CEUS in differentiating the benign and malignant cervical lymph nodes (14), whereas few studies reported its application in the differential diagnosis of head and neck lymphoma and malignant cancerous lymph nodes.

In this study, we focused on the diagnosis value of CEUS in the differentiation of head and neck lymphomas and cancerous lymph nodes. Contrast-enhanced ultrasonography imaging and parameters of cervical lymph nodes were examined, and receiver operating characteristic (ROC) curve analysis was performed.

## Materials and Methods

### Patients

This study was reviewed and approved by the ethics committee (West China Hospital Biomedical Ethics Association). The inclusion criteria included the following: (1) patients with cervical lymphadenopathy admitted to West China Hospital, Sichuan University, Chengdu, China, from November 2015 to August 2017; (2) aged 18–80 years; (3) a cervical lymph node biopsy or surgical resection biopsy was planned. The following patients were excluded from the study: (1) pregnant or in lactation; (2) allergy to contrast agent; (3) patients with severe heart failure, coronary heart disease, or pulmonary hypertension; (4) patients with other contraindications to CEUS; (5) patients who have been diagnosed with lymphoma or have received treatment. Finally, 143 patients were included in this study. Written informed consent was signed by all patients before the examination.

### CEUS Examination

An ultrasound scanner (iU22; Philips Healthcare, Bothell, WA, USA) with an L9-3 linear array transducer was used to perform the CEUS examination, and the frequency was set to 3–9 MHz. Contrast agent SonoVue (Bracco, Milan, Italian) was added with 0.9% NaCl solution and shaken thoroughly to form a milky microbubble suspension before use. The TIC analysis was performed using the software of QLAB (Phillips, Amsterdam, Netherlands).

Patients were supine, and the cervical region was fully exposed; the cervical lymph nodes and surrounding tissue were scanned longitudinally, laterally, and obliquely. Then, the imaging mode was switched to CEUS examination mode. Two-dimensional display function was used to facilitate the contrast observation of lesions, and the nodules were fixed in the largest section. A bolus of 2.4-mL well-equipped contrast agent microbubble suspension was injected intravenously (right cubital vein, 20-gauge cannula) and flushed with 10 mL 0.9% saline solution (NaCl). The built-in timer of the ultrasonic diagnostic instrument started at the same time, and the angiography process was continuously observed in real time for at least 3 min. During this period, patients were told to breathe calmly and keep the posture as constant as possible. After completion of the angiography process, the dynamic video was played back.

Contrast-enhanced ultrasonography images were, respectively, reviewed by two physicians who have been engaged in ultrasound diagnosis for more than 5 years. Controversial cases were reanalyzed and discussed until a unified conclusion was achieved. Characteristics of the lymph nodes shown in the CEUS image include (1) lesion's enhancement order: due to the different directions of contrast agent, enhancement order of lymph nodes includes centrifugal perfusion and centripetal perfusion; (2) internal lesion's homogeneity: according to whether the contrast agent filled the defect area within the lesion at the time of peak, internal lesion's homogeneity was divided into homogeneous enhancement and inhomogeneous enhancement; (3) lesion's enhancement degree: compared with adjacent tissues around the lymph node region at peak enhancement, the degree of nodal enhancement could be divided into hyperenhancement, hypoenhancement, and no enhancement; (4) presence of perfusion defects: whether there is a filling defect area after the injection of contrast agent (15).

Then, a region of interest (ROI) was selected to perform the quantitative analysis using Philips QLAB quantification software. Typically, the lymph node region showing high perfusion was selected as ROI (mostly located in the cortex near the capsule), with an average size of 25 mm^2^. Main parameters provided by the software's automatic tracing TIC included the derived peak intensity (PI), time to peak intensity (TP), and area under the curve (AUC).

### Histopathologic Diagnosis

In order to obtain a histopathologic diagnosis, the patient underwent a fine-needle aspiration biopsy or a surgical biopsy. According to the pathological results, nodules were divided into lymphomas or malignant metastatic lymph nodes. Results of CEUS examination were compared with pathological diagnosis.

### Statistical Analysis

SPSS 22.0 software (IBM Corp., Armonk, NY, USA) was used for data analysis. The CEUS quantitative parameters PI, TP, and AUC were all represented as mean ± SD. Two independent-samples *t*-test was used to compare the numerical results, and categorical parameters were compared by χ^2^. Regression analysis was used to perform the combined diagnosis of image characteristics and parameters. Receiver operating characteristic curve was used to analyze the diagnostic valve of CEUS in distinguishing head and neck lymphoma from malignant nodes. *P* < 0.05 was considered to be statistically significant.

## Results

In total, 143 cases (76 males and 67 females) were included in our research; the average age was 53.4 ± 12.9 years (range, 24–79 years). Both CEUS examination and pathological diagnosis (surgical excision or needle biopsy) were performed in these cases, with their surgical history or histological examination results in the past analyzed. Pathologic results show that 63 of the 143 enlarged lymph nodes were lymphomas, and 80 were metastases (66 cases of nasopharyngeal carcinoma, 7 cases of tonsil cancer, 4 cases of epulis, 2 cases of breast cancer, and 1 case of sarcoma).

**Figure 1:**
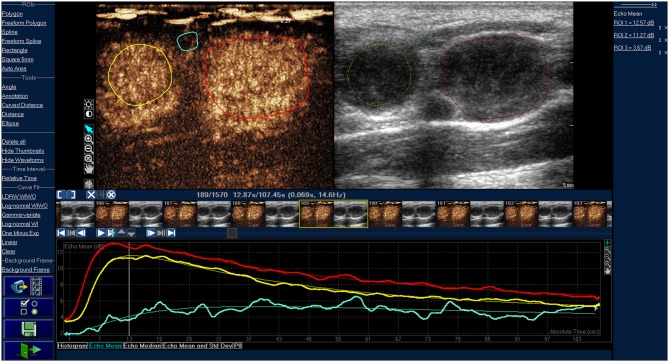
Time-intensity curve of typical cervical lymphoma. The red and yellow ROIs are lymphomas, the red and yellow line are corresponding TIC curves. The blue ROI is the tissue around the lesions, and the blue line is the corresponding TIC curve. The lymphomas were characterized by homogeneous enhancement.

**Figure 2:**
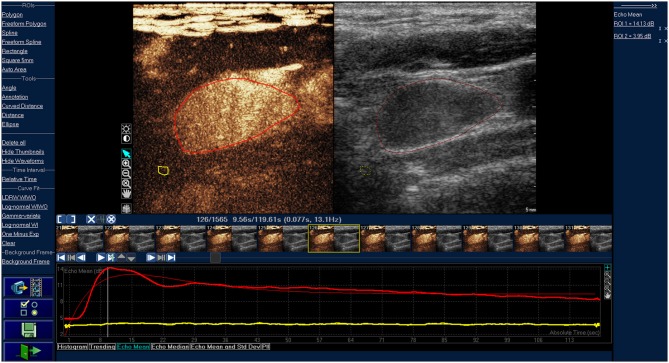
Time-intensity curve of typical cancerous lymph nodes. The red ROI is cancerous lymph nodes, and the red line is corresponding TIC curve. The yellow ROI is the tissue around the lesion, and the yellow line is the corresponding TIC curve. The cancerous lymph node was presented as inhomogeneous enhancement.

Characteristics of CEUS images in lymphoma and cancerous lymph nodes are compared in Table 1, and representative images are shown in Figure 1 (lymphoma) and Figure 2 (cancerous node). Internal homogeneity of lymphoma was very different from that of cancerous lymph nodes: the majority of lymphomas showed homogeneous enhancement patterns (45/63, 71.43%), whereas most of cancerous lymph nodes were inhomogeneous enhancement (66/80, 82.50%) (*P* = 0.020). Besides, centripetal perfusion accounts for the majority (74/80, 92.50%) of cancerous nodes, whereas in lymphoma the proportion of centripetal perfusion was approximately only half (37/63, 58.73%) (*P* = 0.000). In terms of enhancement degree, there were no significant differences between lymphoma and cancerous lymph nodes. Most CEUS images, in both lymphomas (61/63, 96.83%) and cancerous lymph nodes (78/80, 97.50%), were hyperenhancement (*P* = 0.808). Presence of perfusion defects was more common in the cancerous nodes (30/80, 37.50%) than in lymphomas (5/63, 7.94%) (*P* = 0.000), and a ring-enhancing margin was observed in the cancerous nodes only (2, 2.50%) (*P* = 0.504).

**Table 1:** CEUS image characteristics in cervical lymphoma and cancerous lymph nodes.

**Characteristics of CEUS image**	**Lymphomas (*n* = 63)**	**Cancerous lymph nodes (*n* = 80)**	***P*-value**
Lesion's enhancement order			0.000
Centrifugal perfusion	26 (41.27%)	6 (7.50%)	
Centripetal perfusion	37(58.73%)	74 (92.50%)	
Internal lesion's homogeneity			0.020
Homogeneous enhancement	45 (71.43%)	14 (17.50%)	
Inhomogeneous enhancement	18 (28.57%)	66 (82.50%)	
Lesion's enhancement degree			0.808
Hyper-enhancement	61 (96.83%)	78 (97.50%)	
Hypo-enhancement	2 (3.17%)	2 (2.50%)	
No enhancement	0	0	
Presence of perfusion defects			0.000
Yes	5 (7.94%)	30 (37.50%)	
No	58 (92.06%)	50 (62.50%)	
Ring high enhancement of surrounding area			0.504
Yes	0	2 (2.50%)	
No	63 (100%)	78 (97.50%)	

Comparisons of CEUS parameters in two groups are shown in Table 2. Peak intensity of metastasis (10.51 ± 2.98) was higher than that of lymphomas (8.78 ± 2.53) (*P* = 0.000). Likewise, AUC of malignant lymph nodes (784.09 ± 340.24) was higher than that of lymphomas (652.62 ± 249.60) (*P* = 0.009). There was no significant difference in TP between cancerous lymph nodes and lymphomas: 16.51 ± 6.95 in cancerous lymph nodes vs. 16.20 ± 5.77 in lymphomas (*P* = 0.776).

**Table 2:** Quantitative CEUS Findings in cervical lymphoma and cancerous lymph nodes.

**Group**	**No. of cases**	**PI, dB**	**TP,s**	**AUC, dB × s**
Lymphomas	63	8.78 ± 2.53	16.20 ± 5.77	652.62 ± 249.60
Cancerous lympho nodes	80	10.51 ± 2.98	16.51 ± 6.95	784.09 ± 340.24
*P*-value		0.000	0.776	0.009

**Figure 3:**
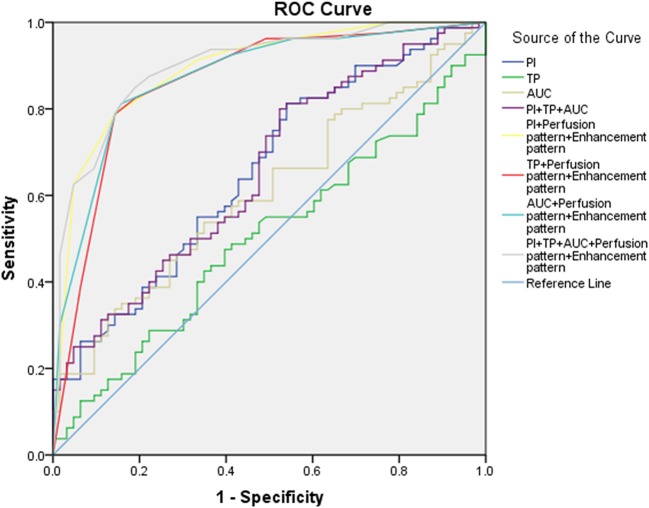
ROC curve for the assessment of the diagnostic value of CEU.

An ROC curve was performed to assess the accuracy of CEUS in the differential diagnosis of head and neck lymphoma and cancerous lymph node of the neck (Table 3, Figure 3). According to the ROC curve, the sensitivity of PI in the diagnosis of head and neck lymphomas and metastases was high (81.25%, with a cutoff value of 8.47). But we should notice that the diagnostic accuracy of these parameters was not so high. Considering that CEUS image characteristics of lymphoma differ greatly from cancerous lymph nodes, we combined the CEUS parameters with the images to fully determine the diagnostic value of CEUS. The results demonstrate that the combination of images and parameters brought more satisfactory sensitivity, specificity, and accuracy (85.00%, 80.95%, and 0.899, respectively).

**Table 3:** ROC analysis:lymphoma vs. cancerous nodes.

**Index**	**AUROC value**	***P*-value**	**CI 95%**	**Cut-off value**	**Sensitivity (%)**	**Specificity (%)**
PI	0.657	0.0006	0.567–0.746	8.47	81.25	46.03
TP	0.501	0.9821	0.406–0.596	11.03	73.75	15.87
AUC	0.606	0.0249	0.514–0.697	935.27	32.5	87.3
PI+TP+AUC	0.655	0.0007	0.565–0.744		80	47.62
PI+Perfusion pattern+Enhancement pattern	0.893	<0.0001	0.84–0.947		78.75	85.71
TP+Perfusion pattern+Enhancement pattern	0.863	<0.0001	0.799–0.927		78.75	85.71
AUC+Perfusion pattern+Enhancement pattern	0.874	<0.0001	0.815–0.933		81.25	84.13
PI+TP+AUC+Perfusion pattern+Enhancement pattern	0.899	<0.0001	0.847–0.95		85	80.95

## Discussion

Studies have reported the clinical application of CEUS in the differential diagnosis of benign and malignant lymph nodes. Cui et al. (16) pointed out that CEUS had a potential diagnostic value in distinguishing tuberculous lymph nodes from metastatic lymph nodes. In line with this, Yu et al. (14). suggested that CEUS was more accurate than conventional ultrasonography in the detection of malignant lymphadenopathy in superficial lymph nodes. Also, there have been several reports that described the usefulness of CEUS in the detection of lymphoma. The findings of Xin et al. (17) showed that CEUS may allow the evaluation of the therapeutic response in lymphoma by detecting the superficial enlarged lymph nodes in lymphoma, and Wei et al. (18) summarized the imaging characteristics of primary thyroid lymphoma in CEUS. However, the diagnostic value of CEUS in the differential diagnosis of head and neck lymphoma and cancerous lymph nodes was never discussed. Clinical symptoms of head and neck lymphoma are similar to those of cancerous lymph nodes, whereas their treatments and prognosis are significantly different, which make it crucial to diagnose the two types of lymphadenopathy accurately (2). Our results suggest that, based on the imaging pattern and quantitative analysis, CEUS may hopefully be one of the excellent imaging techniques for differentiation of head and neck lymphoma and cancerous lymph nodes.

According to our research, inhomogeneous enhancement was observed in the majority of malignant metastases in CEUS, whereas lymphomas mostly manifested as homogeneous enhancement. This is related to angiogenesis and vascular distribution in the lesions. Because of the rapid growth rate of the tumor, immature neovascularization, and avascular necrosis areas are common in cancerous metastatic lymph nodes, which impedes the distribution of contrast agent to these areas, thus resulting in perfusion defects, whereas in most of the lymphomas, blood vessels are highly hyperplastic, which allows the contrast microbubbles flow easily and distribute to the whole lymphoma rapidly to show homogeneous enhancement.

As for enhancement order, our results show that centripetal perfusion was more common in cancerous lymph nodes than in lymphomas. This was consistent with the view of most scholars who believe that metastasis of malignant tumors started from the edge of nodes through the peripheral lymphatic vessels and subsequently caused the perfusion of contrast agent from the periphery to the center (19). It is worth mentioning that malignant cancerous lymph nodes usually maintained the same CEUS perfusion pattern as the primary tumor, which provided a basis for judging whether the cervical lymphadenopathy was lymph node metastasis from the primary tumor or a new lymphoma.

Although not common, the ring high enhancement of surrounding area in metastasis was observed in our study, but none was observed in lymphoma. In line with this, a study believed that the peripheral subcapsular vessel was a typical feature of cancerous lymph nodes (19). The explanation was that unlike lymphoma, which appeared in lymph nodes and progressed in a centrifugal manner in lymph nodes, cancerous nodes entered lymph nodes through afferent lymphatic vessels and spread from the marginal sinuses.

Although CEUS image features play an important role in the differential diagnosis of head and neck lymphoma and cancerous metastatic lymph nodes, it still has certain limitations. For radiologists, it takes a long learning process before they can make a diagnostic report for CEUS examination. In addition, because of the large subjectivity, the judgment results among different inspectors are inevitably divided. Therefore, it is very important to find a more objective evaluation method, for example, to provide diagnostic basis according to CEUS image parameters. According to the research of Yuan et al. (20) the parameters obtained from CEUS examination have important diagnostic value for breast cancer and can distinguish the homogeneity and heterogeneity of tumors. As far as we know, there is still no article to differentiate head and neck lymphoma from cancerous metastatic lymph nodes according to CEUS parameters. In our study, quantification analysis of the CEUS data was performed.

There are mainly three types of parameters in CEUS. One type of parameters is related to blood volume, such as PI, which indicates the maximum dose of contrast agent filling the ROI within a certain period of time. The larger the blood volume, the more contrast agent arriving in the region after being injected into the blood vessel, the higher the PI. The other is time related, such as TP, which represents the time required for PI to reach the highest intensity. The third parameter, such as AUC, is determined by the above two parameters. In this research, PI, TP, and AUC were selected as the main parameters to be studied. According to the results, PI and AUC of the cancerous cervical lymph node were higher than those of lymphoma (*P* < 0.001), whereas TPs of the two groups were similar. This indicates that the blood volume of cancerous metastatic lymph nodes is richer than that of head and neck lymphoma, and PI and AUC could offer a novel clinical perspective in identifying them. However, we should also note that the contrast enhancement of lymph nodes may be affected by many other factors, including the specific administration of the contrast agent and the parameters of the scanner and the metabolism of patients. In addition, the selection of the ROI could directly influence the data used for quantitative analysis. Therefore, the value of CEUS parameters in the diagnosis of lymphoma and cancerous lymph nodes remained to be further studied.

According to the ROC curve analysis, the parameters of CEUS were highly sensitive in the differential diagnosis of head and neck lymphoma and malignant metastatic lymph nodes. Therefore, the parameters of CEUS may be of value in the diagnosis of head and neck lymphoma. However, we should note that a high diagnostic accuracy was not achieved by relying solely on the CEUS parameters. Satisfactory diagnostic results were obtained when CEUS parameters and images were combined. This suggests that when using CEUS to diagnose cervical lymph nodes it is necessary to combine the image characteristics and parameters of nodes to make a comprehensive judgment and draw a reasonable conclusion.

Our study does have some limitations. For example, the number of cases included in this study was not large enough. Additionally, the classification of specific lymphoma was not performed, and the CEUS images or parameters of various lymphomas were not compared.

## Conclusion

Our results suggest that the use of CEUS examination was efficient in the differential diagnosis between lymphoma and malignant metastatic lymph nodes. However, further investigations with large number of cases are needed to explore the role of CEUS in the differentiation of lymphoma and cancerous nodes before this approach can be clinically applied.

## Data Availability Statement

The raw data supporting the conclusions of this article will be made available by the authors, without undue reservation, to any qualified researcher.

## Ethics Statement

The studies involving human participants were reviewed and approved by West China Hospital, Sichuan University. The patients/participants provided their written informed consent to participate in this study. Written informed consent was obtained from the individual(s) for the publication of any potentially identifiable images or data included in this article.

## Author Contributions

The conception and design of the study: WL, JN, and XM. Acquisition of data: WL and JN. Analysis and interpretation of data: QY, HJ, and XO. Drafting the article: JN and WL. The article and final approval of the version to be submitted: XM.

## Conflict of Interest

The authors declare that the research was conducted in the absence of any commercial or financial relationships that could be construed as a potential conflict of interest.
